# Knowledge, Attitude and Perception Regarding Artificial Intelligence in Periodontology: A Questionnaire Study

**DOI:** 10.7759/cureus.48309

**Published:** 2023-11-05

**Authors:** Ruhee L Chawla, Nidhi P Gadge, Sunil Ronad, Alka Waghmare, Aarti Patil, Gargi Deshmukh

**Affiliations:** 1 Periodontics, Jawahar Medical Foundation ACPM Dental College, Dhule, IND; 2 Prosthodontics, Jawahar Medical Foundation ACPM Dental College, Dhule, IND; 3 Dentistry, Deshmukh Dental Clinic, Aurangabad, IND

**Keywords:** questionnaire survey, prognosis, treatment planning, periodontist, artificial intelligence

## Abstract

Introduction: The utilization of artificial intelligence (AI) and machine learning (ML) models has brought about a significant transformation in the manner in which periodontists gather information, evaluate associated risks, develop diverse treatment alternatives, anticipate and diagnose dental conditions that compromise periodontal health. The principal objective of this prospective study was to examine periodontists’ understanding and acceptance of the application of AI in the realm of periodontology.

Materials and methods: This observational study was conducted on 275 participants based on questionnaire using Google Forms. These forms were pre-validated and subsequently circulated among periodontists in Maharashtra via various social media platforms. The study, in its entirety, comprised four open-ended questions on participants’ demographics and 14 closed-ended questions, all of which were presented to the participants in English. These questions aimed to elicit participants' awareness, knowledge, attitudes, and perspectives regarding emerging applications of AI in the field of periodontology. To analyze the collected data, researchers employed the widely utilized Statistical Package for Social Sciences (SPSS) version 22.0.

Result: A 75% response rate was achieved and 68% of the respondents were female. 62% periodontists were aware of AI; however, only 24% were aware of its working principles. Most respondents agreed with the use of AI in periodontal diagnosis; however, they disagreed with the use of AI in predicting clinical attachment loss (69%). 80-82% respondents felt that AI should be a part of postgraduate training and should be implemented in clinical practice. However, most periodontists do not use AI for diagnostic or research purposes. 49% periodontists felt that AI does not have better diagnostic accuracy than periodontists, and therefore cannot replace them in the future.

Conclusion: Most periodontists possessed a reasonable level of understanding regarding the utilization of AI in the domain of periodontology and expressed a desire to incorporate it into their diagnostic and treatment planning processes for periodontal conditions. Additional endeavors must be undertaken to enhance periodontists’ awareness concerning the effective implementation of AI within their professional practice, with the aim of facilitating personalized treatment planning for their respective patients. It is postulated that the integration of AI will augment the likelihood of achieving favorable outcomes within the realm of periodontology.

## Introduction

Artificial intelligence (AI) refers to the imitation or improvisation of human intelligence in machines specifically designed and programmed for numerous cognitive functions, such as problem solving. An exemplary transformation occurs in almost every industry and sector owing to advancements in machine learning (ML) and deep learning (DL) [1].

In 1955, the term ‘artificial intelligence’ was introduced by John McCarthy, a mathematician widely known as the father of AI. This term was chosen to elucidate the machine’s capability to perform tasks that could be interpreted as “intelligent” activities [2]. AI has become crucial to clinical decision-making, quick and authentic data interpretation, the efficiency of computerized data in reducing activities unrelated to patient care, and patients’ education and motivation to improve their health. In dentistry, AI has been proposed for the digitalization of dental records. These records are helpful for automating anatomical landmarks, identifying diseases, and diagnosing tumors. The potential applications of AI in dentistry are rapidly advancing [3].

AI programs can significantly benefit novice periodontists. A convolutional neural network (CNN), used as an unsupervised diagnostic tool, allows periodontists to view computed tomography dental images to facilitate the accurate diagnosis of periodontal diseases and the detection of plaque, gingivitis, and implant design systems [4].

Despite the numerous advantages of AI, its application in periodontics remains markedly restricted. This can be attributed to a myriad of factors, such as the lack of comprehensive familiarity among periodontists with the underlying principles of AI as well as their limited awareness regarding its true potential domains. Furthermore, the general population is reluctant to place trust in the outcomes offered by AI in the context of robot-assisted surgery. Consequently, a multitude of challenges persist and necessitate a confrontational resolution.

Many studies and reviews have reported on the use of AI in periodontics, but no study has been conducted to date to assess the current knowledge and perception of periodontists on the use of AI for periodontal diagnosis and treatment planning [5-9]. Therefore, the present study aimed to analyze periodontists’ knowledge and acceptance of the application of AI in periodontology.

## Materials and methods

Study design

A cross-sectional questionnaire study was conducted with periodontists in Maharashtra to assess their knowledge, attitudes, and perceptions of the use of AI in the field of periodontology. The data were collected over a period of one month from March 2023 to April 2023. This study was approved by the Institutional Ethical Committee of the Jawahar Medical Foundation ACPM Dental College, Dhule (EC/NEW/INST/2022/2959/Y23/212). This study was conducted in accordance with the ethical standards of the Declaration of Helsinki. Participants were granted the opportunity to complete the form on a single occasion. Subsequently, participants' responses were collected after obtaining their informed consent and willingness to participate in the study as well as to elucidate the objective and ensure the preservation of anonymity.

Study area and population

The study was conducted in the Department of Periodontics, Jawahar Medical Foundation ACPM Dental College, Dhule, on the faculty members who were working as periodontists in the dental colleges of Maharashtra state. 

Inclusion and exclusion criteria

Periodontists employed as faculty members who agreed to participate in the study were included. Undergraduates, postgraduates pursuing dental education, private practitioners, and participants who did not give their consent were excluded from the study.

Sample size estimation

The sample size for the online survey was calculated using GPOWER (version 3.1) software developed by Franz Faul at the University of Kiel in Germany. The formula used for analysis was (Z-score)^2^ × standard deviation (SD) x (1-SD) / (margin of error)^2^. The confidence level was 95% and the margin of error was 5%. The standard deviation of the unknown population was 50%. The expected sample size was 207. The study was conducted with 275 periodontists, considering a 30% non-response rate of emails.

Sampling and data collection procedure

The selection of respondents for the study was accomplished using convenience and non-probability sampling. A link to an online survey was created using Google Forms and distributed among periodontists in Maharashtra via 42 WhatsApp groups (one WhatsApp group per college) between March and April 2023. The examiner explained the study objectives to the participants and they were given a brief introduction to the AI. Participants were asked to select one option from the answers provided to each question. Interested participants entered their names and contact information on Google Forms. Responses were made on a single webpage with a “submit” button that allowed only one submission through the link. This study aimed to examine respondents’ approach to AI and its possible future in periodontology. The repeated reminders were sent every five days for one month to complete the forms.

Testing the validity and reliability of questionnaire

The questionnaire was formed in consultation with six experts: three periodontists, two AI experts, and one researcher with more than 10 years of experience who were not involved in the study. Following the assessment conducted by these six specialists, Aiken's V statistic was derived, exhibiting a value of 0.85, signifying favorable content validity. A meticulously crafted survey was developed to accomplish the research objectives, drawing on the content validity. To evaluate the dependability of the inquiries, a preliminary examination or pretesting of the questionnaire was carried out on 40 individuals who were not involved in the study. The reliability of the questionnaire was tested using Cronbach’s alpha, which was 0.88. The questionnaire was retested after a period of two weeks using the same cohort to assess the level of agreement among the questions. Inter-observer agreement was assessed using kappa coefficient, which was 0.96.

Tools and technique

The survey served as the primary instrument for the data collection. The questionnaire was divided into four sections. The first section, known as Part A, focused on four open-ended questions on sociodemographic characteristics, where participants entered their name, age, gender, academic affiliation, and institution in which they are currently working. Part B consisted of seven closed-ended questions, identifying the basic knowledge of the periodontists participating in AI using a Likert three-point scale (agree, neutral, disagree) [10]. Part C consisted of four questions assessing the attitude of periodontists towards the use of AI (two questions used a Likert three-point scale, and two questions used a dichotomous scale of yes or no). Part D consisted of three questions focusing on the perception of periodontists regarding AI application using a three-point Likert scale.

Statistical analysis

Data obtained from the questionnaires were entered into an Excel spreadsheet to serve as a database. The acquired data were subjected to statistical analysis using the SPSS software version 23 (SPSS for Windows, Chicago, USA). The Shapiro-Wilk test was used to assess the normal distribution of the data. Frequency distributions and tables were used to summarize and present the sociodemographic variables and participants' responses. To determine the significance between variables, non-parametric tests, such as the Chi-square test, were employed. Spearman’s correlation coefficient test was also used to assess the relationship between gender, and designation with knowledge, attitude, and perception. The level of significance was set at P ≤0.05.

## Results

Demographic details of the respondents

The study involved 275 participants, of whom 207 individuals filed the online Google Form, generating a response rate of 75%. Descriptive analysis showed that 68% of females and 32 % of males responded to the questionnaire. Of the responded periodontists, 28% were professors, 33 % were readers, and 39% were senior lecturers. 73% of the respondents were in the age group of 25-40 years, as shown in Table 1.

**Table 1:** Demographic characteristics of the periodontists of Maharashtra (n=207)

Variable	Category	n (%)
Age group (in years)	25-30	81 (37.5)
	31-40	70 (36.1)
	41-50	38 (18.1)
	51-60	18 (8.3)
Designation	Professor	56 (27.8)
	Reader	67 (33.3)
	Senior lecturer	84 (38.9)
Gender	Female	138 (68.1)
	Male	69 (31.9)

Response assessing knowledge of periodontist about AI and its applications

A total of 129 periodontists (62%) were aware of the term 'artificial intelligence' with a statistically significant difference (p<0.05); however, only 55 (24%) of them were aware of the working principle of AI with no statistically significant difference (p>0.05). Non-significant differences were noted when participants were asked about the use of AI for diagnosing periodontal bone, aggressive and chronic periodontitis, predicting the prognosis of periodontally compromised teeth (PCT), predicting the prognosis of treatment, and clinical attachment levels (p>0.05). However, most of the periodontists agreed with the use of AI in diagnosing periodontal problems, except for the prediction of clinical attachment level, where 69% of respondents disagreed, and assessment of prognosis of PCT, where 51% respondents gave a neutral response (Table 2).

**Table 2:** Comparison of response frequencies of the participants according to the designation using Chi-square test AI: Artificial intelligence; PCT: Periodontally compromised teeth *p value<0.05: Significant

Knowledge-based questions	Response	Senior lecturer	%	Reader	%	Professor	%	Total (%)	p value
You are aware of term 'artificial intelligence'.	Agree	63	75	43	64	23	41	129 (62)	0.002*
	Neutral	19	23	22	33	30	54	71 (34)	
	Disagree	2	2	2	3	3	5	7 (4)	
You are aware of the working principal of AI.	Agree	22	26	14	21	15	27	51 (24)	0.596
	Neutral	32	38	34	51	25	45	91 (44)	
	Disagree	30	36	19	28	16	29	65 (32)	
AI can be used in clinical diagnosis of periodontal bone loss.	Agree	67	80	51	76	42	75	160 (77)	0.562
	Neutral	14	17	11	16	8	14	33 (16)	
	Disagree	3	4	4	6	6	11	13 (7)	
AI can diagnose cases of aggressive periodontitis and chronic periodontitis.	Agree	76	90	59	88	45	80	180 (87)	0.483
	Neutral	6	7	5	7	8	14	19 (9)	
	Disagree	2	2	3	4	3	5	8 (4)	
AI can assess the prognosis of PCT.	Agree	34	40	27	40	32	57	93 (45)	0.08
	Neutral	48	57	38	57	20	36	106 (51)	
	Disagree	2	2	2	3	4	7	8 (4)	
AI can predict the prognosis of treatment provided in periodontal problems.	Agree	56	67	45	67	40	71	141 (68)	0.679
	Neutral	22	26	20	30	12	21	54 (26)	
	Disagree	6	7	2	3	4	7	12 (8)	
AI can predict the clinical attachment levels.	Agree	23	27	18	27	10	18	51 (25)	0.705
	Neutral	5	6	3	4	4	7	12 (6)	
	Disagree	56	67	46	69	42	75	144 (69)	
Attitude-based questions									
AI should be an integral part of postgraduate training.	Agree	75	89	52	78	42	75	169 (82)	0.193
	Neutral	5	6	10	15	8	14	23 (11)	
	Disagree	4	5	5	7	6	11	15 (7)	
AI based software should be used in clinical practice for ease in radiographic analysis of periodontal diseases.	Agree	67	80	56	84	43	77	166 (80)	0.247
	Neutral	12	14	6	9	12	21	30 (14)	
	Disagree	5	6	5	7	1	2	11 (6)	
Have you used AI for research purpose in periodontics?	Yes	34	40	20	30	30	54	84 (40)	.023*
	No	50	60	47	70	26	46	123 (60)	
Have you used AI in diagnosing periodontal problems?	Yes	4	5	3	4	4	7	11 (5)	.772
	No	80	95	64	96	52	93	196 (95)	
Perception-based questions									
AI has better diagnostic ability than periodontists.	Agree	25	30	20	30	10	18	55 (27)	0.441
	Neutral	19	23	15	22	18	32	52 (25)	
	Disagree	40	48	32	48	28	50	100 (48)	
AI can replace periodontist in future.	Agree	32	38	15	22	12	21	59 (29)	0.049*
	Neutral	20	24	12	18	14	25	46 (22)	
	Disagree	32	38	40	60	30	54	102 (49)	
AI can be a beneficial tool in situations like Covid-19.	Agree	70	83	52	78	45	80	167 (81)	0.698
	Neutral	12	14	10	15	8	14	30 (14)	
	Disagree	2	2	5	7	3	5	10 (5)	

Response assessing attitude of periodontists about AI

82% periodontists felt that AI should be a part of postgraduate training, and 80% felt that it should be used in clinical practice for ease of radiographic analysis of periodontal diseases with non-significant differences (p>0.05). 60% periodontists did not use AI for research purposes, and this difference was statistically significant (p<0.05). 95% periodontists did not use AI to diagnose periodontal problems (p>0.05), as shown in Table 2.

Response assessing perception of periodontists towards AI

48% respondents disagreed with the better diagnostic ability of AI than periodontists, with a non-significant difference (p>0.05), and 49% of respondents disagreed that AI could replace periodontists in the future, with a statistically significant difference (p<0.05). 81% periodontists felt that AI can be a beneficial tool in pandemic situations such as Covid-19 with a non-significant difference (p>0.05), as shown in Table 2.

Correlation between knowledge, attitude, and perception with gender and designation of the participants

The knowledge, attitude, and perception of the participants demonstrated a weakly favorable correlation with gender and designation, indicating that older males, with more years of experience and higher job titles, possessed a greater understanding, mindset, and interpretation regarding the utilization of AI and its applications in the field of periodontology. Knowledge displayed a moderately positive correlation with attitude and a strongly positive correlation with perception, revealing that as periodontists' understanding of AI increased, their mindset and interpretation also improved, as shown in Table 3.

**Table 3:** Correlation between knowledge, attitude, and perception with gender and designation of the participants using Spearman's Correlation Coefficient test *r = 0-0.19: Weak correlation; **r = 0.40-0.59: Moderate correlation; ***r = 0.60-0.79: Strong correlation; ****r = 1: Monotonic correlation

Variables	Gender	Designation	Knowledge	Attitude	Perception
Gender	1****				
Designation	0.165*	1****			
Knowledge	0.063*	0.012*	1****		
Attitude	0.093*	0.067*	0.55**	1****	
Perception	0.016*	0.023*	0.67***	0.43**	1****

## Discussion

AI has a vast array of medical applications and has recently experienced a notable surge in its prevalence, thus necessitating a thorough exploration of its implementation in the field of dentistry, particularly periodontology. The integration of AI into the realms of Medicine and Dentistry has been facilitated by the advent of smartphones and internet connectivity, aligning it with the latest advancements in engineering and technology. Nevertheless, numerous scientists and medical practitioners remain unfamiliar with AI and its potential ramifications in both their personal and professional lives [6]. To the best of our knowledge, this survey stands out because of its distinctive focus on the assessment of periodontists’ knowledge, attitude, and perception regarding the application of AI. To date, no surveys have been conducted in this domain.

The study's findings revealed that the response rate reached 78%, with the majority of the participants being female (68%). This observation can potentially be attributed to the higher number of females pursuing dentistry as their chosen profession, with a significant portion opting for higher education [11,12]. The attitude, knowledge, and perception of periodontists are important for the successful performance of AI in periodontal healthcare, as scant information has been reported to date. The results of this study showed that periodontists were aware of the use of AI in the field of periodontology and had basic knowledge and interest in learning about AI and its applications.

Additionally, most periodontists agree with the use of AI in determining periodontal bone loss, and diagnose cases of aggressive or chronic periodontitis. The majority of them were senior lecturers who agreed, which aligns with the findings of previous studies [4,7,8]. Papantonopoulos et al. and Devito et al. concluded that AI can effectively be used to categorize patients with aggressive or chronic periodontitis based on their immune response profiles [13,14]. In contrast, 20% periodontists agree, and 33% were neutral regarding the role of AI in predicting the prognosis of PCT. According to Lee et al., AI can accurately diagnose and predict the extraction of PCT [15].

However, most of the periodontists disagree with the use of AI to predict clinical attachment levels. Scott et al. conducted a scoping review on the use of AI in periodontics, and concluded that there was lot of heterogeneity in data obtained from the studies, leading to varying results [16]. AI models employed in the research should be evaluated by impartial authorities using genuinely unfamiliar data. Nonetheless, none of the studies compared the results obtained from an entirely autonomous panel of evaluators using unfamiliar data, in contrast to the suggested AI/ML results. Labelling the images for training and subsequent reference tests is also of utmost significance in the field. Several studies have implemented gold standards to ensure the accuracy and reliability of this procedure. This involved the involvement of multiple clinicians or a specialist radiologist in performing the manual tasks, thereby establishing a consensus-based approach. Nevertheless, it is worth noting that, in certain studies, a single evaluator was responsible for performing this task. This approach, although potentially introducing a single-operator bias, may have reduced the external validity of the results. Additionally, the internal validity of the findings could have been compromised because of the potential introduction of systematic error [16]. According to Miller et al., the accuracy of AI in detecting periodontal bone loss and clinical attachment levels ranged from as low as 25% to 60% [17].

The present study revealed that most senior lectures were aware of and had knowledge about AI compared to readers and professors. The reason for this phenomenon could potentially be attributed to the escalated level of contact that young individuals have with digitization, which encompasses their inclination to investigate and gain knowledge about novel technological advancements, such as AI [18].

The knowledge, attitude, and perception about the use of AI in diagnosing periodontal problems was found to increase with the increase in designation or level of experience of the periodontists. However, most of them did not use AI in their dental practice or for research purposes. This finding is in agreement with previous studies [6,18,19]. This might be due to lack of awareness, lack of training in colleges, cost, and concerns regarding the potential for errors in the AI system that may not be easily identifiable, resulting in erroneous therapeutic decisions [18,20].

82% respondents felt that AI should be a part of their postgraduate curriculum and should be implemented in clinical practice. This finding is supported by previous studies [17,18,19]. Periodontitis presents a significant challenge for healthcare professionals in accurately identifying and diagnosing this condition. The current standard of care emphasizes the evaluation of soft tissues through the utilization of a graduated probe, while the assessment of hard tissues is accomplished through radiographic imaging. Nevertheless, these approaches suffer from suboptimal inter- and intra-operator reliability, primarily because of the inherent variability associated with probing pressure and radiographic angulation [21]. The investigation of periodontitis is intricate and multifaceted owing to the presence of a multitude of factors that contribute to its manifestation and progression. The examination of these factors necessitates the utilization of AI, a cutting-edge technological tool that can unravel the complex interplay between these factors and provide a comprehensive understanding of their impact on the process of diagnosing the disease or comprehending its underlying causes [1].

49% respondents disagree that AI can replace periodontists in diagnosing periodontal diseases, but they feel that AI can successfully help in taking their dental practice to a new level. This is in accordance with previous studies [19,21,22]. AI is currently unable to replicate and reproduce the complex and multifaceted nature of a doctor-patient relationship through the implementation of an intellectually captivating discourse characterized by genuine empathy, a profoundly uplifting demeanor, and a genuine interest in fostering a sense of trust and confidence between medical professionals and patients. Second, the integration of medical history with the physical examination process in situations characterized by ambiguity and uncertainty is beyond the current reach of AI technology, rendering it unfeasible and impracticable.

Recommendations

To enhance and foster the growth and motivation of periodontists, it is important to integrate AI into the dental curriculum or regular training programs. To expand our knowledge and understanding in this field, it is recommended that future research endeavors concentrate on the development of models that possess a heightened level of accuracy when it comes to diagnosing various dental ailments, as well as predicting the outcomes of diverse treatment methods. Periodontists should use AI for diagnosis, and treatment planning in their practice. 

Limitations of the study

The present study did not identify any barriers to AI use in periodontology. The sample was not representative of postgraduates or private practitioners. Closed-ended questions utilizing Likert scale may have hindered the generation of suggestions or proposals for questions requiring multiple perspectives, thereby leading to miscommunication. Future surveys should be conducted on samples of diverse populations, including postgraduates, private practitioners, and open-ended questions, where participants can express their views on AI.

## Conclusions

Periodontists have varying opinions on AI applications. Therefore, it is important to understand that AI is not a suitable substitute for periodontists and dental professionals. Instead, it would be helpful for better diagnosis, prognosis, and treatment plans in periodontology. The prevailing number of respondents possessed knowledge regarding the advantages associated with the utilization of AI in periodontology and held the conviction that it would serve as a valuable resource. This study revealed that enhanced technological provisions within dental clinics and the instruction of practitioners at both the undergraduate and postgraduate levels could potentially overcome forthcoming obstacles pertaining to the incorporation of AI in the field of periodontology.
